# Sulfonated Amphiphilic Poly(α)glutamate Amine—A Potential siRNA Nanocarrier for the Treatment of Both Chemo-Sensitive and Chemo-Resistant Glioblastoma Tumors

**DOI:** 10.3390/pharmaceutics13122199

**Published:** 2021-12-20

**Authors:** Adva Krivitsky, Sabina Pozzi, Eilam Yeini, Sahar Israeli Dangoor, Tal Zur, Sapir Golan, Vadim Krivitsky, Nitzan Albeck, Evgeny Pisarevsky, Paula Ofek, Asaf Madi, Ronit Satchi-Fainaro

**Affiliations:** 1 Department of Physiology and Pharmacology, Sackler Faculty of Medicine, Tel Aviv University, Tel Aviv 69978, Israel; advashy@gmail.com (A.K.); sabina.pozzi26@gmail.com (S.P.); eilamyeini@gmail.com (E.Y.); sahardang@gmail.com (S.I.D.); kwaifeh@gmail.com (T.Z.); sapir5050@gmail.com (S.G.); nitzan.albeck@gmail.com (N.A.); jalchemic@gmail.com (E.P.); Paula.Ofek@gmail.com (P.O.); 2 School of Chemistry, The Raymond and Beverly Sackler Faculty of Exact Sciences, Tel Aviv University, Tel Aviv 69978, Israel; vadimkri777@gmail.com; 3 Sagol School of Neuroscience, Tel Aviv University, Tel Aviv 69978, Israel; 4 Department of Pathology, Sackler Faculty of Medicine, Tel Aviv University, Tel Aviv 69978, Israel; asafmadi@tauex.tau.ac.il

**Keywords:** nanocarrier, polyplexes, siRNA delivery, glioblastoma therapy, amphiphilic poly(α)glutamate, P-selectin

## Abstract

Development of chemo-resistance is a major challenge in glioblastoma (GB) treatment. This phenomenon is often driven by increased activation of genes associated with DNA repair, such as the alkyl-removing enzyme O^6^-methylguanine-DNA methyltransferase (MGMT) in combination with overexpression of canonical genes related to cell proliferation and tumor progression, such as Polo-like kinase 1 (Plk1). Hereby, we attempt to sensitize resistant GB cells using our established amphiphilic poly(α)glutamate (APA): small interfering RNA (siRNA) polyplexes, targeting Plk1. Furthermore, we improved brain-targeting by decorating our nanocarrier with sulfonate groups. Our sulfonated nanocarrier showed superior selectivity towards P-selectin (SELP), a transmembrane glycoprotein overexpressed in GB and angiogenic brain endothelial cells. Self-assembled polyplexes of sulfonated APA and siPlk1 internalized into GB cells and into our unique 3-dimensional (3D) GB spheroids inducing specific gene silencing. Moreover, our RNAi nanotherapy efficiently reduced the cell viability of both chemo-sensitive and chemo-resistant GB cells. Our developed sulfonated amphiphilic poly(α)glutamate nanocarrier has the potential to target siRNA to GB brain tumors. Our findings may strengthen the therapeutic applications of siRNA for chemo-resistant GB tumors, or as a combination therapy for chemo-sensitive GB tumors.

## 1. Introduction

Glioblastoma (GB) is the most common and the deadliest type of malignant primary brain tumor in adults, with an annual age-adjusted incidence rate of 4.4 per 100,000 population. GB patient prognosis is extremely poor, with a 5-year survival rate of only 5.5% [1]. The current treatment regimen includes maximal surgical resection, followed by radiation and chemotherapy with alkylating agents, such as temozolomide (TMZ) [2]. This regimen increases patients’ median survival of 3 to 14 months [3,4]. Complete surgical removal is nearly impossible, due to the invasive nature of GB tumors; therefore, tumor relapse frequently occurs. Recurrence developed in more than 90% of patients within several years, and often displays enhanced resistance to initial chemotherapy treatment [5]. One of the most investigated mechanisms for TMZ chemotherapy-resistance in GB is the activation of the O^6^-methylguanine–DNA methyltransferase (MGMT) enzyme. This enzyme, highly active in GB tumors, is responsible for removing the methyl group from the O^6^ position in guanine nucleotides, thus resolving DNA damage induced following TMZ treatment. The lack of DNA methylation directs the cells to DNA damage repair and rescues them from apoptosis. The activity of the MGMT enzyme can be “switched off” by inducing methylation on the MGMT promoter itself. High levels of MGMT promoter methylation have been shown to correlate with prolonged survival in TMZ treated-GB patients [6]. Therefore, targeting the MGMT gene may sensitize tumor cells to TMZ and increase treatment efficiency in TMZ-resistant patients [7,8,9]. In order to investigate the naturally developed resistance towards TMZ, we have established a resistant clone of U251 human GB cells by culturing the U251 TMZ-sensitive cells (referred to in here as “U251”) in the presence of increasing doses of TMZ for a period of over 6 months (referred to in here as TMZ resistant or “TMZ-R”). Nonetheless, treatments targeting alternative cellular pathways which can potentially overcome TMZ-resistance are still lacking. Gene therapy in general, and RNA therapies in particular, have emerged as a promising approach for the downregulation of oncogenic pathways in GB [10,11,12,13]. In particular, RNA interference (RNAi) is known for its ability to selectively silence upregulated genes at the mRNA level. Among the oncogene-activated pathways that sustain GB progression, we focused on Polo-like kinase 1 (Plk1), traditionally not linked to upregulation of TMZ-resistance genes, which may however attenuate GB aggressiveness and may provide an alternative to GB patients that do not respond to TMZ [14]. This serine/threonine kinase has a pronounced role in mitotic regulation and cell cycle progression. It is overexpressed in various cancers, including GB, and associated with poor prognosis and high risk of cancer metastasis [15,16,17]. We have previously shown that silencing the expression of Plk1 efficiently reduced tumor growth rate and prolonged survival of mice bearing ovarian cancer [18]. In addition, in a mouse model of pancreatic ductal adenocarcinoma (PDAC), Plk1 silencing was shown to synergize with microRNA 34a (miR-34a), via the downregulation of the activated pathway, resulting in tumor growth inhibition [19]. Systemic delivery of RNAi is known to be challenging since short oligonucleotides (OLN) are subjected to rapid renal clearance, degradation while circulating in the body, poor cellular penetration, short half-life, and aggregation in the blood [20,21,22]. In order to overcome these hurdles, a delivery system needs to be developed. Using a well-designed nanocarrier enabled enhanced tumor accumulation, taking advantage of the enhanced permeability and retention (EPR) effect [23,24]. This phenomenon, observed in the tumor vasculature, is defined by high permeability which allows extravasation of macromolecules, as opposed to the tightly bound healthy endothelium. Furthermore, impaired lymphatic drainage associated with cancer may increase drugs accumulation [23]. We have previously developed a polymeric nanocarrier based on poly(α)glutamic acid (PGA), a biodegradable and water-soluble polymer further modified with physiologically-protonated amine groups. Electrostatic-based complexation with the negatively-charged siRNA formed a defined population of nanoparticles with the ability to internalize into cancer cells via endocytosis [25] and induce gene silencing [26]. Furthermore, we introduced a modification with alkyl groups forming inner hydrophobic core exposing the positively-charged moieties outwards in a micellar-like structure [26,27]. Our active polymeric micelles demonstrated biocompatibility and preferential accumulation in several tumor types when systemically injected, leading to an anticancer therapeutic effect. Furthermore, these micelles selectively delivered siRNA to mammary adenocarcinoma tumors without accumulating in the liver and crossed the dense stroma of PDAC, internalizing into tumor cells and achieving silencing of the target [19]. However, delivery of drugs to the central nervous system (CNS) is extremely difficult since the brain is protected by a highly selective mechanism of capillaries, called the blood–brain barrier (BBB) [28]. Even though brain tumors often display compromised BBB and permeable endothelium, the latter varies with tumor type, location, and prior treatment [29]. The inflamed cerebral endothelium often expresses P-selectin (SELP) protein, a transmembrane adhesive glycoprotein that facilitates leukocytes and platelets adhesion [30]. We have previously shown that SELP enhances GB progression by promoting tumor cell proliferation and invasion and immunosuppression via microglia/macrophages anti-inflammatory polarization [31,32]. Recently, we have shown that targeting SELP by the addition of sulfonate moieties on dendritic polyglycerol nanocarrier facilitated nanoparticles accumulation into tumors and enhanced the anticancer activity of Paclitaxel (PTX) in a mouse model of GB [33]. Therefore, we have modified our previously designed amphiphilic poly(α)glutamate amine (APA) [19,27] with sulfonate groups, in order to mimic the tyrosine sulfate moieties of P-selectin glycoprotein ligand-1 (PSGL-1), the natural ligand of SELP [34], and improve drug targeting to the brain. This chemical modification resulted in increased accumulation of the polyplexes into 3D spheroids of U251 TMZ-sensitive or -resistant cells, compared to untargeted nanocarrier. These results highlight the great potential of our nanocarrier as a parenteral injectable, non-toxic, and efficient delivery vehicle for siRNA, now being evaluated for its potential in reaching brain tumors and reducing GB tumorigenesis. We hypothesize that our treatment can serve as combination therapy for chemo-resistant and chemo-sensitive GB tumors.

## 2. Materials and Methods

### 2.1. Materials

All chemicals and solvents were A.R. or HPLC grade. Chemical reagents were purchased from Merck (White House Station, NJ, USA) and Sigma–Aldrich (St. Louis, MO, USA). O-benzyl protected glutamic acid (H-Glu(OBzl)-OH) was purchased from Chemimpex (Dillon Drive, IL, USA). HPLC grade solvents were purchased from BioLab (Jerusalem, Israel). Dulbecco’s modified Eagle medium (DMEM) and fetal bovine serum (FBS) were purchased from Gibco (Crofton Rd, Dún Laoghaire, Dublin, Ireland). All other tissue culture reagents were purchased from Biological Industries Ltd. (Kibbutz Beit Haemek, Israel). MGMT siRNA (siMGMT) was purchased from Ambion (Invitrogen, Waltham, MA, USA). Plk1 siRNA (siPlk1) and green fluorescent protein (GFP) siRNA (siGFP) were purchased from Biospring (Frankfurt am Main, Germany). Cy5-labeled negative control siRNA (Cy5-siNC) was purchased from Sigma–Aldrich (St. Louis, MO, USA). TMZ was purchased from Petrus chemicals (Herzeliya, Israel).

### 2.2. Cell Culture

Glioblastoma (GB) cell lines. Human U251 cells were purchased from the European Collection of Authenticated Cell Cultures (ECACC). Murine GL261 cells were purchased from the National Cancer Institute (Frederick, MD, USA). All GB cells were cultured in DMEM supplemented with 10% FBS, 100 µg/mL of streptomycin, 12.5 U/mL of nystatin, 100 U/mL of penicillin, and 2 mM of L-glutamine. All cells were tested for mycoplasma with a mycoplasma detection kit (Biological Industries Ltd., Beit Haemek, Israel). Cells were grown at 37 °C; 5% CO_2_.

#### 2.2.1. Establishment of mCherry and iRFP Stably-Expressing U251 Cells

mCherry/iRFP infecting viral particles were produced as described previously [35,36]. Briefly, HEK 293T cells were transfected with pQC-mCherry [35] and with the compatible packaging plasmids pCMV-VSV-G (#8454, Addgene, Watertown, MA, USA) and pUMVC (#8449, Addgene, Watertown, MA, USA), or with pLVX-iRFP [37] together with pCMV-VSV-G (#8454, Addgene, Watertown, MA, USA) and psPAX2 (#12260, Addgene, Watertown, MA, USA). Viral particle-containing media were collected after 48 h. For cell infection, U251 cells were incubated with viral particles and polybrene (8 μg/mL) (hexadimethrine bromide) for 8–16 h prior medium replacement. Following 48 h, mCherry-infected cells were selected by puromycin and iRFP infected cells by hygromycin.

#### 2.2.2. Establishment of mCherry U251 Chemo-Resistant Cell Line (U251 TMZ-R)

U251 cells were cultured with increasing doses of temozolomide (TMZ 1 μM to 10 μM) over a period of 6 months.

### 2.3. IC_50_ Determination Assay

U251 or U251 TMZ-R cells were plated onto 96-well plates at a density of 2.5 × 10^4^ cells/well. Twenty-four hours later, cells were treated with TMZ at increasing concentrations (0.001–1000 μM). Following 5 days, cells were counted using Beckman Coulter counter (Beckman Coulter Life Sciences, Indianapolis, IN, USA). Results are presented as the percentage of confluence compared to untreated cells.

### 2.4. RNA Isolation and Quantitative Real-Time RT-PCR (qRT-PCR)

RNA was isolated using EZ-RNA II total RNA isolation kit (Biological Industries, Kibbutz Beit Haemek, Israel) according to the manufacturer’s protocol. Actin was used for normalization as a house-keeping gene. Primers used: PLK1 FOR:5′-CACAGTTTCGAGGTGGATGT-3′, PLK1 REV:5′-ATCCGGAGGTAGGTCTCTTT-3′. MGMT FOR:5′-GTTTGCGACTTGGTACTTGG-3′, MGMT REV:5′-TGCCCAGGAGCTTTATTTCG-3′. Actin FOR: 5’-CCAACCGCGAGAAGATGA-3’, Actin REV: 5’-CCAGAGGCGTACAGGGATAG-3’.

### 2.5. Animal Models

In order to assess Plk1 and SELP expression in GB tumors by histology, 6-week-old male SCID mice (Envigo CRS, Ness-Ziona, Israel) were anesthetized by ketamine (150 mg/kg) and xylazine (12 mg/kg) injected intraperitoneally (IP). Then, iRFP-labeled U251 human GB cells (5 × 10^4^ cells) were stereotactically implanted into the brain striatum (N = 10) as previously described [32]. Tumor development was followed by MRI (MR Solutions Ltd., Guildford, UK) and Maestro™ imaging system (CRI Inc., Woburn, MA, USA) as previously described [10].

#### Animals Ethics Statement

Animals were housed in the Tel Aviv University animal facility. All experiments received ethical approval by the animal care and use committee (IACUC) of Tel Aviv University (approval no. 01-19-097, Approval and expiry dates 15 December 2019–15 December 2023) and conducted in accordance with NIH guidelines.

### 2.6. Frozen OCT Tissue Fixation

Tumor-bearing mice were anesthetized by IP injection of ketamine (100 mg/kg) and xylazine (12 mg/kg), followed by PBS perfusion and 4% paraformaldehyde (PFA). Brains were harvested, then incubated with 4% PFA for 4 h and with 0.5 M of sucrose (BioLab Ltd., Jerusalem, Israel) for 1 h, and 1 M of sucrose for overnight (ON). Brains were embedded in optimal cutting temperature (OCT) compound (Scigen, Thermo Fisher Scientific, Waltham, MA, USA) on dry ice, then stored at −80 °C.

### 2.7. Immunostaining

OCT-embedded tumor samples were cryo-sectioned (5 μm thick sections), and stained using BOND RX autostainer (Leica Microsystems, Wetzlar, Germany). Cryo-sections were immunostained for: Plk1- using rabbit anti-human/mouse Plk1 antibody (Cat. No. BS-3535R dilution 1:50, Bioss Antibodies, Woburn, MA, USA) and Alexa Fluor 488-goat anti-rabbit secondary antibody (Cat. No. ab150077, dilution 1:300, Abcam, Cambridge, UK); SELP- using mouse anti-human SELP antibody (Cat. No. BBA1, Clone BBIG-E, dilution 1:30, R&D systems, McKinley, MN, USA) and Alexa Fluor 568-goat anti-mouse secondary antibody (Cat. No. ab175473, dilution 1:300, Abcam, Cambridge, UK). Slides were blocked by incubating with 10% goat serum in PBS containing 0.02% Tween-20, for 30 min. Then, slides were incubated with primary antibodies for 1 h, washed, and incubated with secondary antibodies for 1 h at room temperature. Then, slides were washed using BOND Wash Solution (Leica Microsystems, Wetzlar, Germany) and treated with ProLong^®^ Gold mounting with DAPI (Thermo Fisher Scientific, Waltham, MA, USA). Then slides were covered with coverslips. Images were obtained using the EVOS FL Auto cell imaging system (Thermo Fisher Scientific, Waltham, MA, USA).

### 2.8. Survival Analysis Based on TCGA Data

For survival analysis we used OSgbm, which assesses the prognostic value of our selected genes [38]. Briefly, OSgbm contains 684 samples with transcriptome profiles and clinical information from The Cancer Genome Atlas (TCGA), Gene Expression Omnibus (GEO), and Chinese Glioma Genome Atlas (CGGA). Survival analysis data were presented by Kaplan–Meier (KM) plot with hazard ratio (HR) and log-rank *p* value.

### 2.9. Synthesis of APA-Sulfonate (APAS)

APA was synthesized by conjugating ethylenediamine and hexylamine groups to a poly(α)glutamate backbone via the pending carboxylic acid moieties as was previously described [19,27]. To a solution of APA (130 mg, 0.592 mmol per monomer) in dry dimethylformamide (DMF) (5 mL), tributylamine was added (270 μL, 1.14 mmol, 1.93 equiv. per monomer), and the reaction was left to stir for 20 min. Then, the reaction mixture was stirred for 1.5 h at 25 °C, under Argon atmosphere (Ar_(g)_). Propanesultone (20 µL, 0.237 mmol, 0.4 equiv. per monomer) was added, and the reaction was left to stir for ON under Ar_(g)_. DMF was removed under vacuum. The remaining residue was suspended in water (30 mL). pH was adjusted to 3.0 with HCl, and the remaining solution was dialyzed against water (8 L), freeze-dried, and lyophilized to obtain a white powder at a yield of 70%.

### 2.10. Multi-Angle Static Light Scattering (MALS)

The molecular weight of APA was determined using Agilent 1200 series HPLC system (Agilent Technologies Santa Clara, CA, USA), equipped with a multi-angle light scattering detector (Wyatt Technology, Santa Barbara, CA, USA). APA was separated using Kw404-4F column (Showa Denko America Inc., New York, NY, USA) and a mixture of 0.5 M AcOH in ACN: double distilled water (DDW) 4:6 (*v*/*v*%) as a mobile phase. Sample was prepared at a concentration of 4 mg/mL in the mobile phase buffer, then filtered using a 0.2-μm filter prior to the analysis. Sample was ran at a flow of 0.5 mL/min. Molecular weight (Mw) was analyzed using the ASTRA software (Wyatt Technology, Santa Barbara, CA, USA).

### 2.11. Scanning Electron Microscope (SEM)

APA:siPlk1 sample was prepared in DDW at 0.1 mg/mL concentration (APA equiv.). The sample was dropped on a silicon wafer and allowed to dry. SEM images were obtained using Quanta 200 FEG Environmental SEM (FEI, Hillsboro, OR, USA) at high vacuum and 3.0 KV. Images were collected using secondary electrons detector.

### 2.12. Dynamic Light Scattering (DLS) and Phase Analysis Light Scattering (PALS)

Samples were prepared at a polymer concentration of 0.1 mg/mL in 15 mM of HEPES. Size and zeta potential measurements were made with a Mobius DLS/PALS instrument (Wyatt Technology, Santa Barbara, CA, USA). Sixty µL of the sample was loaded into the dip cell. Data were analyzed according to the isotropic sphere method, and were measured as intensity distribution. Average values were calculated based on 3–5 independent measurements. All measurements were performed at 25 °C.

### 2.13. Elemental Analysis (EDS)

APA and APAS samples were prepared in DDW at a concentration of 10 mg/mL, then, dropped on a silicon wafer, allowed to dry, and dropped again several times. EDS was performed by an LN Oxford thin window detector of 138 eV resolution and ISIS software using Quanta 200 FEG Environmental SEM (FEI, Hillsboro, OR, USA) at high vacuum and 20.0 KV.

### 2.14. Proliferation Assay

U251 and U251 TMZ-R cells were plated onto 96-well plates at densities of 7.5 × 10^4^ and 5 × 10^4^ cells/well, respectively. Twenty four hours later, cells were treated with APA:siPlk1 or APA:siGFP at 5 N/P ratio and 250/500 nM. Following 20 h, cells were imaged using IncuCyte ZOOM™ (Satorius, Goettingen, Germany). Red channel images were taken using a 10× objective. Results were calculated by the IncuCyte™ Software. Results were presented as confluence percentage compared with untreated cells.

### 2.15. Multicellular Tumor Spheroids (MCTS)

MCTS were prepared from GL261 cells, mCherry-labeled U251, or U251 TMZ-R cells by a modified hanging-drop method [33]. Briefly, cells were seeded in droplets (2500 cells/25 μL for staining/internalization assay and 8000 cells/25 μL for flow cytometry analysis) in 4:1 DMEM:methylcellulose. Following 72 h, spheroids were embedded in Cultrex^®^ Reduced Growth Factor Basement Membrane Matrix (R&D systems, McKinley, MN, USA) and incubated for 48 h. The matrix was then dissolved using Cell Recovery Solution (Corning, Watertown, NY, USA) and spheroids were transferred to Eppendorf tubes for further treatment with APA:Cy5-siRNA, APAS:Cy5-siRNA, or Cy5-siRNA alone. For SELP staining, spheroids were incubated with mouse anti-human SELP primary antibody for ON (Cat. No. BBA1, Clone BBIG-E, dilution 1:50, R&D systems, McKinley, MN, USA), then washed and incubated with mouse IgG kappa binding protein (m-IgGκ BP) conjugated to CruzFluor™ 488 (CFL 488) (Cat. No. sc-516176, dilution 1:300, Santa Cruz Biotechnology Inc., Dallas, TX, USA) secondary antibody for 3 h. SELP inhibitor (KF38789, Cat. No. 2748, Tocris BioScience, Bristol, UK) was added 1 h prior to treatment at a concentration of 2 μM to the relevant tubes. For confocal imaging, spheroids were collected, washed, fixed with 4% PFA in PBS solution, and mounted on coverslips. The Leica SP8 imaging system (Leica Microsystems, Wetzlar, Germany) was used for imaging. For FACS analysis, recovered spheroids were dismantled to create a single-cell suspension. Cells were incubated with mouse anti-human SELP primary antibody (BBA1, R&D) for 1 h on ice. Then, cells were washed and incubated with mouse IgG kappa binding protein (m-IgGκ BP) conjugated to CruzFluor™ 488 (CFL 488) (sc-516176, Santa Cruz Biotechnology, Inc., Dallas, TX, USA) for 1 h on ice. Cells were then washed with PBS supplemented with 5 mM of EDTA, 1% sodium azide, and 1% FBS. Fluorescent intensity was analyzed by flow cytometry using Attune NxT cytometer (Thermo Fisher Scientific, Waltham, MA, USA) and analyzed by Kaluza 2.1 software (Beckman Coulter, Brea, CA, USA).

### 2.16. SELP Expression in 3D vs. 2D Cell Culture

To assess SELP expression, iRFP-labeled U251 GB cells were grown in 10-cm^2^ petri-dish (1 × 10^6^ cells) or as 3D spheroids (as detailed above in the MCTS section) for 48 h. Cells from 2D cell culture were harvested using a cell scraper, whereas cell suspension from 3D spheroids was obtained by matrix digestion using Cell Recovery Solution, as previously described. SELP expression was evaluated using flow cytometry analysis as described above.

### 2.17. Infra-Red Spectroscopy

ATR-IR spectroscopy was performed using TENSOR 27 spectrometer (BRUKER, Billerica, MA, USA).

### 2.18. Electrophoretic Shift Assay (EMSA)

Evaluation of polymer:siRNA complexation at N/P ratios between 1 and 25 was performed by mixing 50 pmol of siRNA and APA/APAS polymers at different concentrations in DDW and incubate for 20 min at room temperature. Mobility of free and nanocarrier-complexed siRNA was then analyzed by agarose-gel electrophoresis.

### 2.19. Western Blot

Cells were seeded in 6-well plates at a density of 3 × 10^5^ cells/well. After 24 h, cells were treated with 100 nM of siRNA-equivalent dose. Following 48 h, cells were harvested and lysed using NP40 reagent. Lysates were loaded and ran into a 12% acrylamide gel under 120 V for ~2 h. Proteins were transferred to nitrocellulose membrane under a current of 250 mA for 2 h. The nitrocellulose membrane was blocked with 5% skim milk in TBST (15 mM of NaCl, 1 mM of Trisma base, pH = 8.0, 0.1% Tween 20) for 1 h, and incubated with rabbit MT3.1 anti-MGMT antibody (Abcam, Cambridge, UK) (1:1000 in TBST), or mouse anti-HSC 70 antibody (Cat. Sc-7298, Clone B6, Santa Cruz Biotechnology, Dallas, TX, USA) (1:40,000 in TBST) ON at 4 °C. Secondary horseradish peroxidase (HRP)-conjugated goat anti-rabbit, or goat anti-mouse antibodies (Jackson Immunoresearch, Baltimore, PA, USA; Abcam, Cambridge, UK) were incubated with nitrocellulose membrane at 1:10,000 in TBST for 1 h. Blots were developed using Westar Supernova ECL kit (Cyanagen, Bologna, Italy) in accordance with the manufacturer’s protocol.

### 2.20. H-Nuclear Magnetic Resonance (NMR)

APA/APAS samples were dissolved in deuterium oxide. NMR spectroscopy was obtained using 400 MHz Avance, (Bruker, Billerica, MA, USA).

### 2.21. Internalization of APA/APAS:Cy5-siRNA Polyplexes into U251 Cells (2D)

Cells were seeded (5 × 10^4^ cells/well) on 13-mm cover glasses in a 24-well plate and incubated for 24 h. Cells were treated with 100 nM of siRNA-equivalent concentration alone or complexed with APA/APAS for 20 min, then washed several times with PBS, fixed with 4% PFA for 30 min at room temperature, and washed with PBS again. Cells were then mounted on slides using ProLong^®®^ Gold antifade reagent with DAPI (Thermo Fisher Scientific, Waltham, MA, USA). Internalization was followed using Leica SP8 confocal imaging systems (X60 Magnification) (Leica Microsystems GmbH, Wetzlar, Germany).

### 2.22. Statistical Analysis

Data were presented as mean ± standard deviation (represented graphically as error bars). Statistical significance was analyzed by Student’s *t*-test.

## 3. Results and Discussion

### 3.1. TMZ-Induced Resistance in U251 GB Cells Enhances MGMT mRNA Levels but Does Not Alter Plk1 mRNA Levels

**Figure 1:**
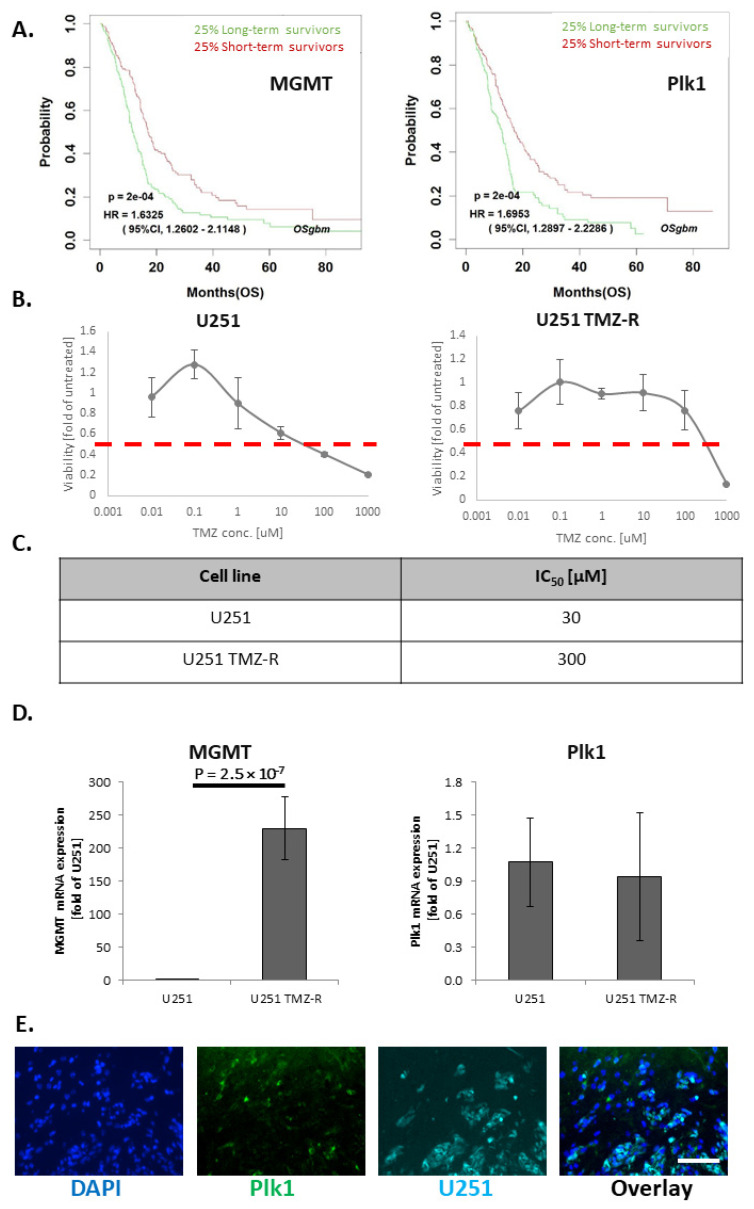
The development of resistance to TMZ enhances MGMT expression without altering Plk1 oncogene levels in GB. (**A**) Survival analysis performed on OSgbm database comparing MGMT expression (left panel) and Plk1 expression (right panel) of the top 25% longest GB survivors versus the bottom 25% shortest survivors. (**B**) Inhibitory concentration 50% (IC_50_) plot of parental U251 glioblastoma cells vs. TMZR clone. (**C**) Table summarizing the IC_50_ of wild-type U251 cells versus U251 TMZ-R cells. (**D**) TMZ resistance in U251 occurred via upregulation of MGMT, without changing Plk1 levels. (**E**) Intracranial U251 GB tumors show Plk1 expression (scale = 100 µm).

To establish a correlation between the expression levels of MGMT or Plk1 and GB patient’s survival, we obtained data from the OSgbm database [38]. Survival data of 684 GB patients (25% long term and 25% short term survivors) revealed that high expression of both MGMT and Plk1 significantly correlated with short-term survival, while low expression of both proteins significantly correlated with long-term survival of GB patients (Figure 1A). In order to investigate the expression levels of these two proteins in the context of TMZ-resistance, we first evaluated the establishment of a TMZ- resistant (U251 TMZ-R) clone. Hence, the proliferation assay performed on U251 and U251 TMZ-R cells showed that cell viability was reduced to 50% following 72 h incubation with 30 and 300 μM TMZ in U251 and U251 TMZ-R cells, respectively (Figure 1B,C). Furthermore, the expression levels of MGMT and Plk1 mRNA in both U251 and the U251 TMZ-R clone were evaluated by real-time qPCR (Figure 1D). The obtained results confirmed that in comparison to parental U251, TMZ-R cells express significantly higher levels of MGMT, while Plk1′s expression remained unchanged following the acquirement of TMZ resistance. Therefore, we concluded that the inhibition of Plk1 could be an additional and attractive strategy to use in combination with TMZ in order to improve the survival of GB patients, whether sensitive or resistant to chemotherapy. Figure 1D shows low expression of MGMT in U251 compared with U251 TMZ-R cells (~230-fold change), while Plk1 expression in both cell lines was similar. Furthermore, cryosection of U251 tumors, obtained in intracranially injected SCID mice, confirmed the expression of Plk1 in our GB mouse model (Figure 1E).

### 3.2. APA:siPlk1 Complexes Efficiently Silence Plk1 Expression in Both U251 and U251 TMZ-R Cells

**Figure 2:**
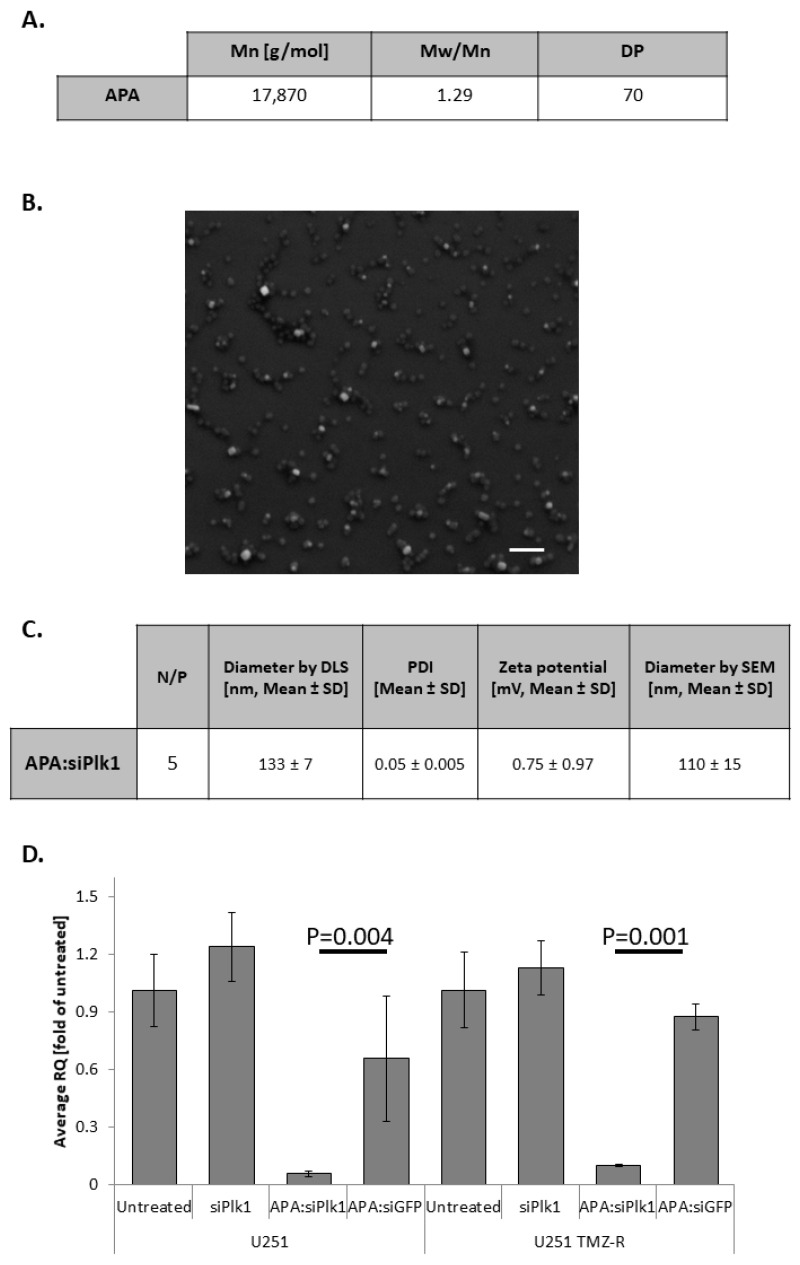
Physico-chemical characteristics and silencing activity of APA:siPlk1 polyplexes. (**A**) Table summarizing the Mw (Mn), PDI (Mw/Mn), and theoretical degree of polymerization (DP) as obtained by multi-angle light scattering (MALS). (**B**) Scanning electron microscope (SEM) image of the dry droplet of polyplexes (scale = 500 nm). (**C**) Table summarizing the hydrodynamic diameter, polydispersity index (PDI) and zeta potential of the main population of polyplexes, obtained by Mobius and PALS instruments, and the average diameter obtained by SEM. (**D**) Specific mRNA silencing obtained by RT-PCR, performed on U251 and U251 TMZ-R GB cells following treatment with APA:siPlk1 polyplexes.

RNAi therapeutics have the potential to silence “undruggable targets” such as Plk1. To overcome the multiple barriers associated with RNAi delivery, we used our previously published APA RNAi nanocarrier [19,27]. MALS analysis showed APA bears the Mw of 17,870 g/mol, ascribed to 70 repeating units, and have Mw/Mn of 1.29 (Figure 2A). APA was complexed with Plk1 siRNA at N/P ratio of 5, to yield a main population of nanoparticles with a hydrodynamic diameter of 133 ± 7 nm (79.3 ± 1.15% of the nanoparticles by intensity distribution) and an almost neutral zeta potential of 0.75 ± 0.97 mV (Figure 2C, Supplementary Materials Figure S1). Furthermore, the polydispersity index (PDI) of this main population was narrow (0.05 ± 0.005). SEM images of the dry droplet of complexes matched the DLS diameter (110 ± 15 nm) and showed spherical morphology. Similar properties were exhibited by APA:siGFP polyplexes, used in this study as a negative control. The hydrodynamic diameter of the main population (82.6 ± 12.5%) of APA:siGFP polyplexes was 115 ± 32 nm, and its zeta potential was 0.52 ± 0.17 mV (Supplementary Materials Figure S1B,C). Polyplexes at the size range of ~10–150 nm were previously shown to benefit from selective accumulation at the tumor site due to the EPR effect [10,18,36]. Elemental analysis of APA:siPlk1 and APA:siGFP polyplexes confirmed that both complexes contained similar weight percentages of nitrogen and phosphorus (15.14 ± 3.14% of nitrogen and 2.8 ± 0.15% phosphorus in APA:siPlk1 and 13.29 ± 3.02% of nitrogen and 3.11 ± 0.2% phosphorus in APA:siGFP, respectively) (Supplementary Materials Figure S1D). These data verified the fact that the same N/P ratio was used for the two polyplexes to treat GB cells. The ability of APA:siPlk1 polyplexes to induce specific gene silencing in both U251 and U251 TMZ-R cells was evaluated at the mRNA level by RT-PCR. Cells were treated with APA:siPlk1, APA:siGFP, or siPlk1 alone at a concentration of 100 nM siRNA for 48 h. APA:siPlk1 reduced Plk1 mRNA level by ~95% in U251 cells and by ~90% in U251 TMZ-R cells compared to untreated cells, while siPlk1 alone did not induce any silencing in both cell lines examined. APA:siGFP did not significantly reduce Plk1 mRNA levels, showing ~35% and ~13% silencing in U251 and U251 TMZ-R cells, respectively (Figure 2D).

### 3.3. Treatment with APA:siPlk1 Polyplexes Reduces the Viability of Both U251 Cells and U251 TMZ-R Clone

**Figure 3:**
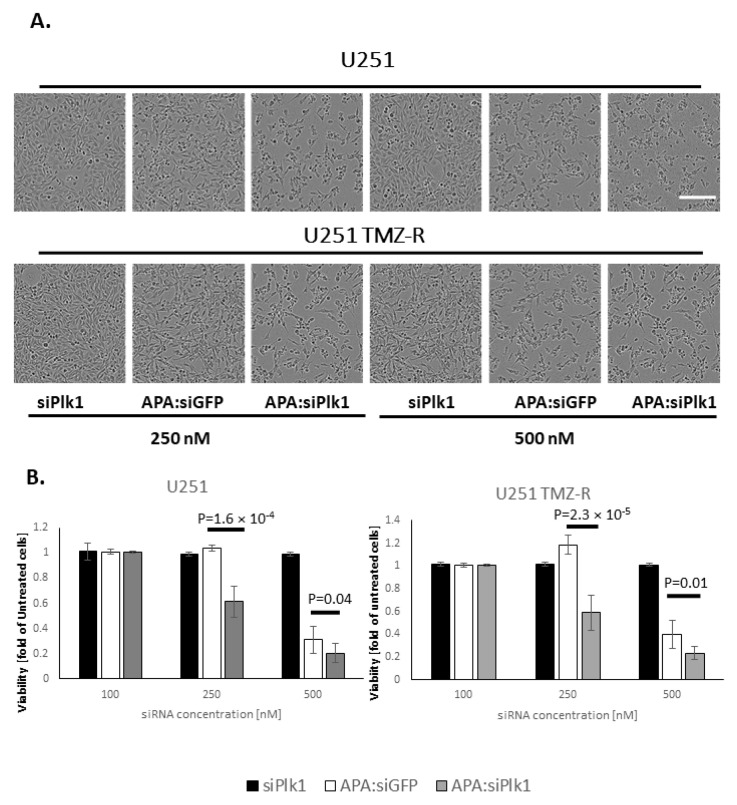
APA:siPlk1 treatment reduced the viability of U251 and U251 TMZ-R cells. (**A**) Representative phase-contrast images of the cells following 20 h of treatment. (**B**) Bar graph of cells viability following 20 h of treatment based on red-channel images (scale = 20 µm).

Next, the effect of treatment with APA:siPlk1 polyplexes on the viability of U251 and U251 TMZ-R cells was evaluated. Cells were treated with APA:siPlk1, APA:siGFP, or siPlk1 alone at siRNA concentrations of 100, 250, and 500 nM for 20 h. Representative images at 20 h are presented (Figure 3A), and bar graphs of cell viability are presented (Figure 3B). While treating U251 and U251 TMZ-R with 100 nM APA:siPlk1, APA:siGFP polyplexes, or siPlk1 alone, did not affect the viability of the cells, 250 nM of APA:siPlk1 polyplexes reduced the viability of U251 and TMZ-R cells by ~40%. On the other hand, treating U251 and U251 TMZ-R with 250 nM APA:siGFP or siPlk1 alone did not cause any cell toxicity. When U251 and U251 TMZ-R cells were exposed to 500 nM of APA:siPlk1 polyplexes, the viability of U251 and U251 TMZ-R cells was reduced by ~80% compared to untreated cells, while siPlk1 alone did not affect the cell viability. Nonetheless, such a high concentration of APA:siGFP polyplexes induced cell toxicity, therefore reducing the cell viability by ~70% and ~60% in U251 and U251 TMZ-R, respectively, compared to untreated cells. The specific and selective activity of APA:siPlk1 polyplexes at 250 nM siRNA equivalent treatment dose demonstrated to be an efficient treatment that affected the proliferation of GB cells.

### 3.4. SELP Is Expressed on the Membranes of GB Cells and Represents a Suitable Candidate for Active Targeting for the Delivery of siRNA Polyplexes

**Figure 4:**
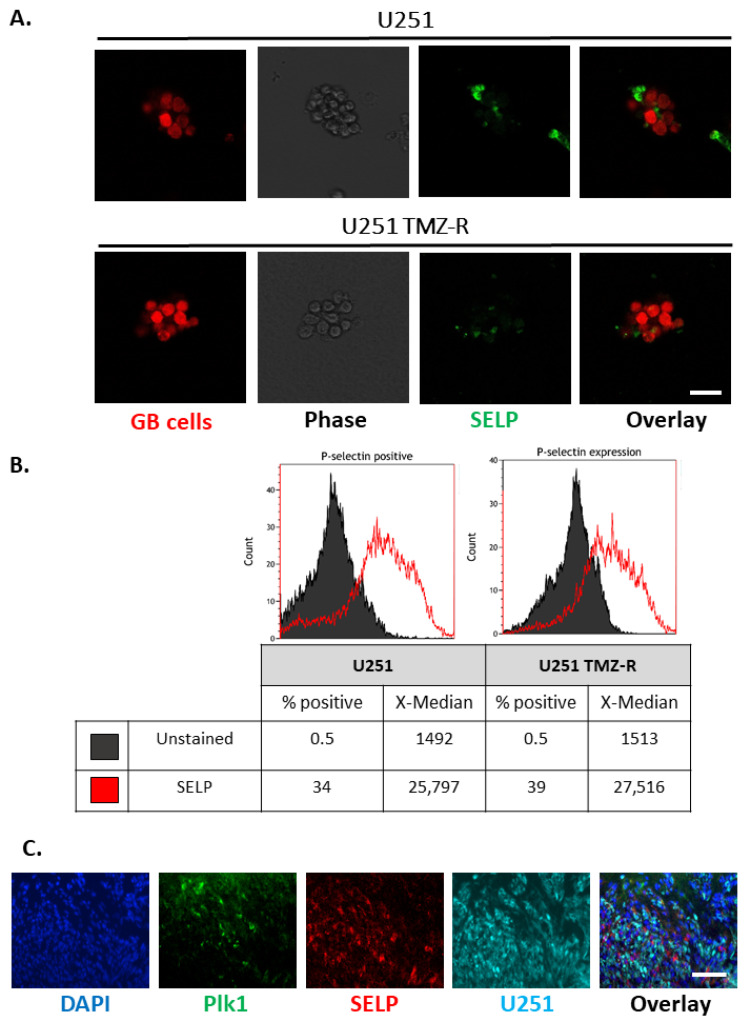
SELP expression on U251 and U251 TMZ-R 3D spheroids. (**A**) Imaged by confocal microscopy (scale = 20 µm) and (**B**) analyzed by fluorescence activated cell sorting (FACS). (**C**) SELP expression on slices of U251 intracranial tumors and co-localization with Plk1 (scale = 100 µm).

SELP was previously found to be upregulated on both GB tumors and inflamed cerebral endothelium at the tumor site, as opposed to its basal expression in healthy brain tissue. Aiming to optimize our APA:siPlk1 by the addition of active targeting, we evaluated the relevance of targeting SELP using U251 GB cells. We first assessed the expression of SELP in U251 and U251 TMZ-R GB spheroids, and in U251 tumor slices (Figure 4). Immunostaining of SELP in U251 and U251 TMZ-R spheroids demonstrated that 3D cultures of GB cells expressed SELP (Figure 4A). Interestingly, flow cytometry analysis for SELP on spheroids of U251 and U251 TMZ-R revealed slightly higher expression of SELP in the TMZ-R cells (34% in U251 compared with 39% positive cells in U251 TMZ-R clone). These findings suggest that targeting SELP would be effective for GB tumors, whether sensitive or resistant to chemotherapy (Figure 4B). Furthermore, flow cytometry analysis for SELP expression in U251 cells grown in 2D monolayer or 3D spheroids demonstrated a remarkable increased expression of SELP in the 3D culture (Supplementary Materials Figure S2A). This highlights the importance and the relevance of using SELP as active targeting for the delivery of our polyplexes to GB tumors [28]. The BBB represents a challenge for the delivery of drugs to the brain. Hence, targeting SELP that is normally expressed on brain endothelium and is upregulated in cancer can facilitate accumulation in GB tumors, as previously observed [33]. Furthermore, the expression of SELP in in vivo settings was validated on slices of U251 intracranial tumors resected from SCID mice (Figure 4C, Supplementary Materials Figure S2B). Immunostaining demonstrated high expression of SELP, nearby to the expression of both the endothelial marker CD31 (Supplementary Materials Figure S2B) and Plk1 (Figure 4C), emphasizing the rationale for targeting SELP in combination with specific Plk1-downregulating therapeutic modality.

### 3.5. Sulfonation of Amphiphilic Poly(α)glutamate Amine (APA)

**Figure 5:**
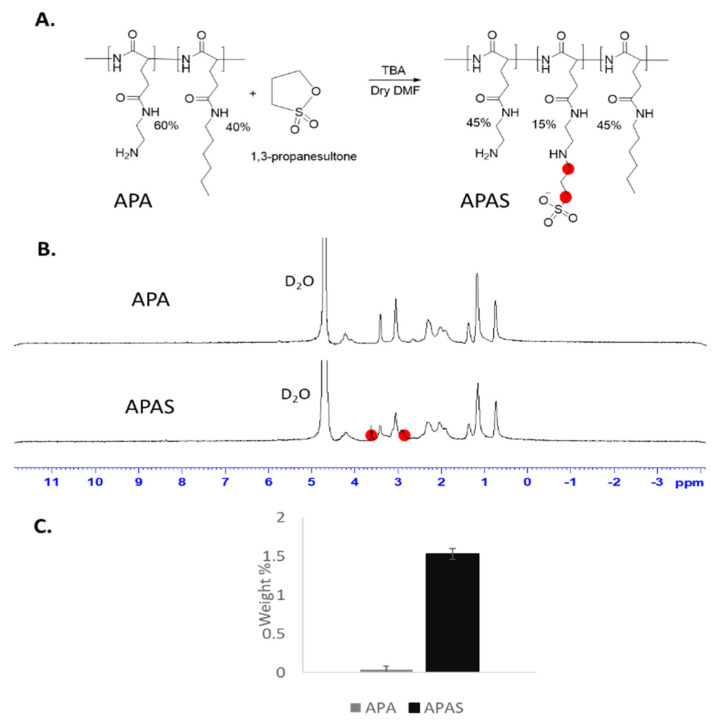
Synthesis and characterization of APAS. (**A**) Modification of APA with sulfonate groups using propanesultone. (**B**) ^1^H-NMR spectrum of APA and APAS in D_2_O. (**C**) Elemental analysis demonstrating the weight percent of Sulfur (S) in dry samples of APA and APAS, as obtained by energy-dispersive X-ray spectroscopy (EDS) analysis.

In order to improve the delivery of our polyplexes to brain tumors, APA was conjugated with sulfonate groups. These groups were shown before to enhance brain uptake and to accumulate in GB tumors due to mimicry of the natural ligand of SELP [33]. APA was modified with sulfonate groups using propanesultone reagent (Figure 5A). Ten equivalents of base (tributylamine) per propanesultone were required for the substitution of 15% of the amine groups. The product was characterized by ^1^H-NMR and the addition of sulfonate groups was validated by the appearance of 2 peaks at the regions of 3.6 and 2.9 ppm (Figure 5B). Furthermore, infrared (IR) spectrum demonstrated the addition of a band at the region of 1000–1050 cm^−1^ characteristic to sulfonic acid group [39] (Supplementary Materials Figure S3). Elemental analysis approved sulfonation ratio of ~15%, corresponding to Sulfur weight % of 1.53 ± 0.07, while APA did not have a detectable amount of Sulfur (0.04 ± 0.04, Figure 5C).

### 3.6. APAS forms Active Complexes with siRNA and Enables Silencing of GB-Relevant Genes

**Figure 6:**
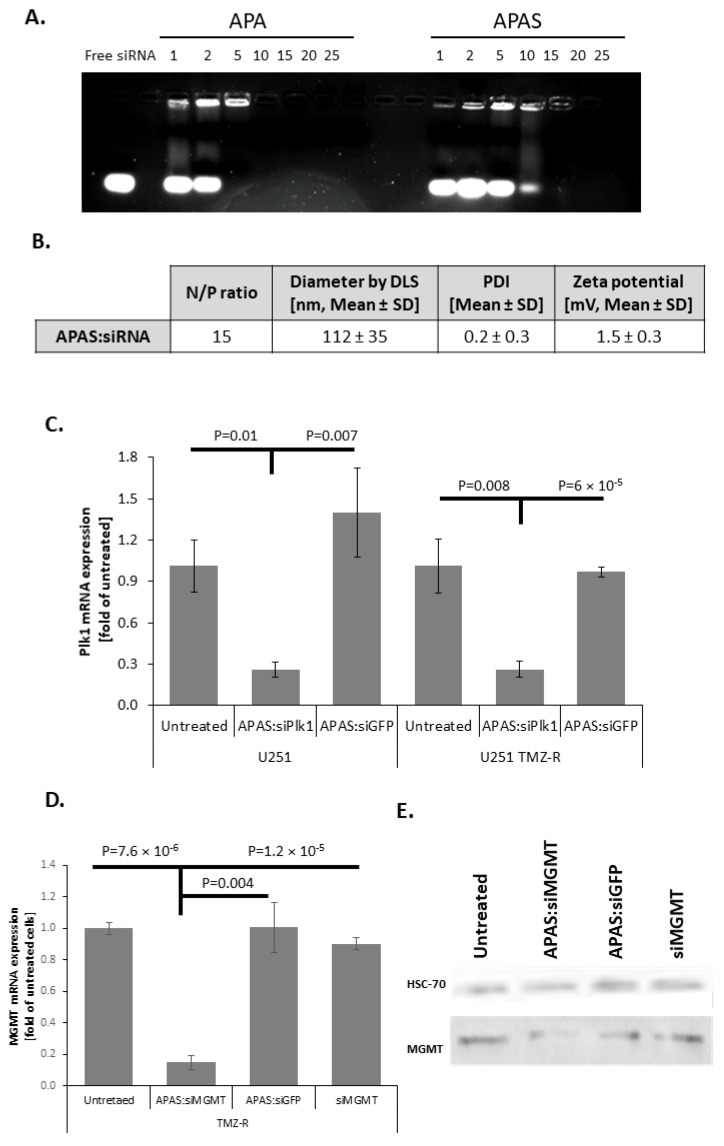
Sulfonate modification maintained size and activity of APA complexes. (**A**) Electrophoretic shift assay (EMSA) of polyplexes of APA and APAS with siRNA at increasing N/P ratios. (**B**) Table summarizing the hydrodynamic diameter, polydispersity index (PDI), and zeta potential of the main population of APAS:siPlk1 polyplexes as obtained by Mobius and PALS instruments. (**C**) Specific Plk1 mRNA silencing obtained by RT-PCR, performed on U251 and U251 TMZ-R GB cells following treatment with APAS:siPlk1 polyplexes. (**D**,**E**) Specific MGMT mRNA silencing obtained by RT-PCR (**D**) and western Blot (**E**) performed on U251 and U251 TMZ-R GB cells following treatment with APAS:siMGMT polyplexes.

We further evaluated complexes formed by self-assembly of APAS and siRNA and compared them with APA:siRNA polyplexes. Hence, we complexed either APA or APAS polymers with siRNA at increasing N/P ratios. Complexes were loaded on an agarose gel supplemented with ethidium bromide, and allowed to run under 100 V for 15 min. Retardation of migration of the free siRNA following neutralization of its negative charge by complexing with the positively charged polymer was evaluated under UV light (Figure 6A). As shown, while APA neutralized the charge of siRNA already at N/P ratio of 5, a higher N/P ratio was required to complex siRNA in the case of APAS. Full complexation between APAS and siRNA was shown only at N/P ratio of 15, due to the extra negative charge of sulfonate groups in physiological pH. Therefore, polyplexes at N/P ratio of 15 were selected for additional characterization and silencing evaluation (Figure 6B,C, Supplementary Materials Figure S4). DLS measurements demonstrated a hydrodynamic diameter of 113 ± 35 nm (80.3 ± 12.5% of the population by intensity distribution) similar to APA:siPlk1 polyplexes, and a slightly higher polydispersity (0.2 ± 0.3) compared to APA:siPlk1 polyplexes. Surface charge was negligibly higher compared to APA:siRNA polyplexes (1.5 ± 0.3 mV), due to the higher N/P ratio used for the complexation of APAS:siRNA polyplexes. Next, selective silencing of APAS:siPlk1 polyplexes was evaluated at the mRNA level in U251 and U251 TMZ-R cells (Figure 6C). While APAS:siGFP did not silence Plk1 mRNA, APAS:siPlk1 reduced Plk1 mRNA levels to ~25% compared to untreated cells in both U251 and U251 TMZ-R cells. To demonstrate silencing of MGMT in TMZ resistant GB cells, U251 TMZ-R cells were treated with APAS:siMGMT, and the mRNA and protein levels were evaluated (Figure 6D,E). The highly MGMT-expressing U251 TMZ-R cells were treated with polyplexes of APAS:siMGMT, APAS:siGFP, or siMGMT alone for 48 h. RT-PCR analysis demonstrated that treatment with APAS:siMGMT reduced the mRNA levels to ~15% of untreated cells, while APAS:siGFP or siMGMT alone did not affect MGMT mRNA levels. Furthermore, western blot analysis corroborated our previous results, showing a reduction of MGMT at the protein level following APAS:siMGMT treatment (Figure 6E).

### 3.7. Sulfonate Modification Facilitated Internalization of Cy5-siRNA into U251 and U251 TMZ-R Spheroids

**Figure 7:**
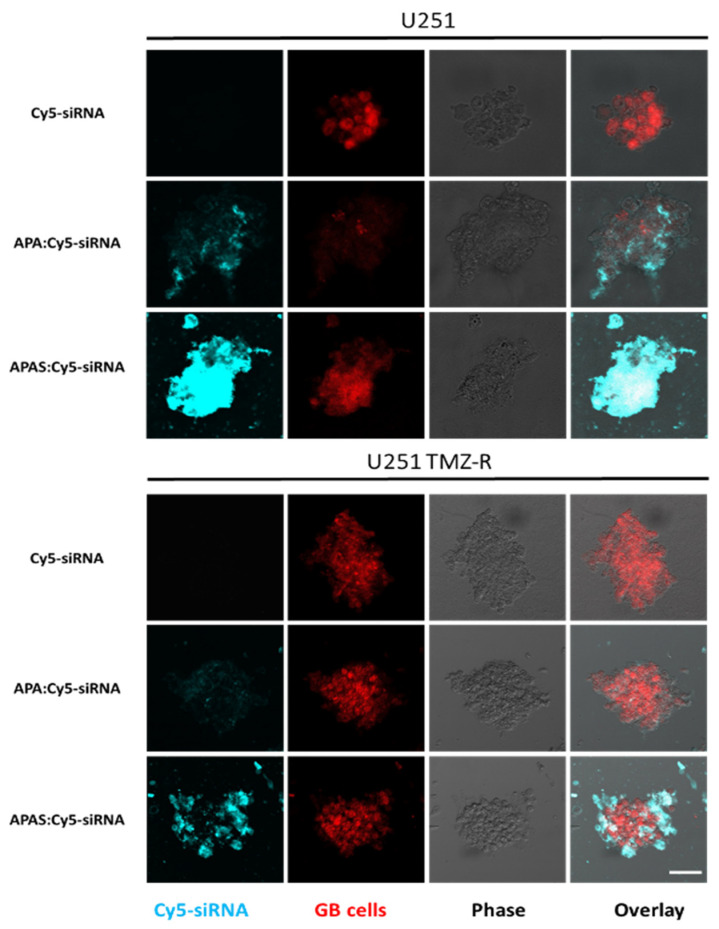
Sulfonate modification of APA facilitated internalization of Cy5-siRNA carrying polyplexes into both U251 and U251 TMZ-R spheroids (scale = 50 µm).

Next, the effect of the SELP targeting on internalization was evaluated using 3D settings of U251 and U251 TMZ-R 3D spheroids. 3D spheroids were treated with APA:Cy5-siRNA, APAS:Cy5-siRNA, or Cy5-siRNA alone for 20 min. While Cy5-siRNA alone was unable to internalize to the 3D spheroids, APA:Cy5-siRNA internalized to both U251 and U251 TMZ-R 3D spheroids. Strikingly, much higher internalization into the 3D spheroids was shown by the APAS:Cy5-siRNA polyplexes (Figure 7). Supplementary Materials Figure S5A shows internalization of APA/S:cy5-siRNA polyplexes into U251 cells grown in 2D culture. As the expression of SELP is much lower in 2D settings compared with the 3D spheroids (Supplementary Materials Figure S2), the effect of targeting SELP in 2D is less pronounced, and the internalization into the cells was only slightly higher following treatment with APAS:Cy5-siRNA compared to APA:Cy5-siRNA (Figure 7). Furthermore, Cy5-siRNA was unable to enter into U251 cells seeded in 2D monolayer as well. To validate the specific targeting of SELP, GL261 murine GB 3D spheroids were disintegrated and treated with either APA:Cy5-siRNA, APAS:Cy5-siRNA or Cy5-siRNA alone in the presence of 2 μM of the SELP inhibitor KF38789 (Supplementary Materials Figure S5B). APAS:Cy5-siRNA polyplexes demonstrated higher internalization into GL261 spheroids-derived cells within 5 min post-treatment (median fluorescence intensity of 3536 compared with 2931, respectively). Furthermore, the addition of SELP inhibitor reduced the internalization of APAS:Cy5-siRNA polyplexes to the level of 2016, while it did not alter the internalization of APA:Cy5-siRNA polyplexes (median fluorescence intensity = 2847, Supplementary Materials Figure S5).

## 4. Conclusions

Resistance to chemotherapy is frequently observed in GB patients that undergo surgical resection and radiotherapy; hence, alternative therapeutic approaches are in need. While targeting signaling pathways that are associated with resistance may improve the outcome of TMZ treatment, silencing oncogenes that are not related to developing chemotherapy resistance such as Plk1, may be an alternative treatment suitable for both chemo-sensitive and chemo-resistant tumors. APA complexed with siPlk1 formed size-controlled, injectable, and non-toxic polyplexes that were able to induce specific gene silencing and affected the proliferation of U251 and U251 TMZ-R cells. In order to maximize the therapeutic benefit, APA was modified with sulfonate groups targeting the siRNA to SELP overexpressed on GB endothelial and tumor cells which led to higher internalization into U251 3D spheroids. Our results highlight the therapeutic potential of sulfonated APA as RNAi nanocarrier, for maximal therapeutic response in chemo-resistant and chemo-sensitive GB brain tumors, which should be further evaluated in vivo.

## Data Availability

The GBM patient sequencing data analyzed are available in the OSgbm database (http://bioinfo.henu.edu.cn/GBM/GBMList.jsp) (accessed on 27 November 2021).

## Supplementary Materials

The following are available online at https://www.mdpi.com/article/10.3390/pharmaceutics13122199/s1, Figure S1: Physicochemical characteristics of APA:siGFP polyplexes. Figure S2: A. High expression of SELP on U251 spheroids (3D) compared with tissue culture (2D) as obtained by fluorescence activated cell sorting (FACS). B. Co-localization of SELP with CD31 in U251 intracranial tumors. Figure S3: IR spectrum of APA and APAS. Figure S4: APAS:siPlk1 representative histogram of the main population of polyplexes as obtained by Mobius DLS instrument. Figure S5: A. Sulfonate modification had minor effect on internalization of Cy5-siRNA carrying polyplexes into U251 cells (2D). B. Inhibition of SELP reduced internalization of APAS:Cy5-siRNA polyplexes into GL261 spheroids, but did not alter the internalization of APA:Cy5-siRNA polyplexes, as obtained by fluorescence activated cell sorting (FACS).

## References

[1] Gittleman H., Boscia A., Ostrom Q.T., Truitt G., Fritz Y., Kruchko C., Barnholtz-Sloan J.S. (2018). Survivorship in adults with malignant brain and other central nervous system tumor from 2000–2014. Neuro-Oncology.

[2] Castro M.G., Cowen R., Williamson I.K., David A., Jimenez-Dalmaroni M.J., Yuan X., Bigliari A., Williams J.C., Hu J., Lowenstein P.R. (2003). Current and future strategies for the treatment of malignant brain tumors. Pharmacol. Ther..

[3] Stupp R., Mason W.P., van den Bent M.J., Weller M., Fisher B., Taphoorn M.J., Belanger K., Brandes A.A., Marosi C., Bogdahn U. (2005). Radiotherapy plus concomitant and adjuvant temozolomide for glioblastoma. N. Engl. J. Med..

[4] Schapira A.H. (2007). Neurology and Clinical Neuroscience.

[5] Rommelaere J., Raykov Z., Grekova S., Kiprijanova I., Geletneky K., Koch U., Aprahamian M. (2018). Oncolytic Virotherapy for Prevention of Tumor Recurrence. U.S. Patent.

[6] Hegi M.E., Diserens A.-C., Gorlia T., Hamou M.-F., de Tribolet N., Weller M., Kros J.M., Hainfellner J.A., Mason W., Mariani L. (2005). MGMT gene silencing and benefit from temozolomide in glioblastoma. N. Engl. J. Med..

[7] Papachristodoulou A., Signorell R.D., Werner B., Brambilla D., Luciani P., Cavusoglu M., Grandjean J., Silginer M., Rudin M., Martin E. (2019). Chemotherapy sensitization of glioblastoma by focused ultrasound-mediated delivery of therapeutic liposomes. J. Control. Release.

[8] Sloan A.E., Roger L., Murphy C., Reese J., Lazarus H.M., Dropulic B., Gerson S.L. (2019). A phase I study of MGMT-P140K transfected hematopoetic progenitor cells (HPC) combined with TMZ/O^6^BG dose escalation for newly diagnosed, MGMT unmethylated glioblastoma: Tolerance and evidence of survival benefit. Am. Soc. Clin. Oncol..

[9] Yu W., Zhang L., Wei Q., Shao A. (2020). O^6^-methylguanine-DNA Methyltransferase (MGMT): Challenges and New Opportunities in Glioma Chemotherapy. Front. Oncol..

[10] Ofek P., Calderon M., Mehrabadi F.S., Krivitsky A., Ferber S., Tiram G., Yerushalmi N., Kredo-Russo S., Grossman R., Ram Z. (2016). Restoring the oncosuppressor activity of microRNA-34a in glioblastoma using a polyglycerol-based polyplex. Nanomedicine.

[11] Tiram G., Ferber S., Ofek P., Eldar-Boock A., Ben-Shushan D., Yeini E., Krivitsky A., Blatt R., Almog N., Henkin J. (2018). Reverting the molecular fingerprint of tumor dormancy as a therapeutic strategy for glioblastoma. FASEB J..

[12] Zafir-Lavie I., Sherbo S., Goltsman H., Badinter F., Yeini E., Ofek P., Miari R., Tal O., Liran A., Shatil T. (2018). Successful intracranial delivery of trastuzumab by gene-therapy for treatment of HER2-positive breast cancer brain metastases. J. Control. Release.

[13] Shatsberg Z., Zhang X., Ofek P., Malhotra S., Krivitsky A., Scomparin A., Tiram G., Calderón M., Haag R., Satchi-Fainaro R. (2016). Functionalized nanogels carrying an anticancer microRNA for glioblastoma therapy. J. Control. Release.

[14] Higuchi F., Fink A.L., Kiyokawa J., Miller J.J., Koerner M.V., Cahill D.P., Wakimoto H. (2018). PLK1 inhibition targets Myc-activated malignant glioma cells irrespective of mismatch repair deficiency–mediated acquired resistance to temozolomide. Mol. Cancer Ther..

[15] Strebhardt K., Ullrich A. (2006). Targeting polo-like kinase 1 for cancer therapy, Nature reviews. Cancer.

[16] Pezuk J., Brassesco M., Morales A., de Oliveira J., Queiroz R.d., Machado H., Carlotti C., Neder L., Scrideli C., Tone L. (2013). Polo-like kinase 1 inhibition causes decreased proliferation by cell cycle arrest, leading to cell death in glioblastoma. Cancer Gene Ther..

[17] Lerner R.G., Grossauer S., Kadkhodaei B., Meyers I., Sidorov M., Koeck K., Hashizume R., Ozawa T., Phillips J.J., Berger M.S. (2015). Targeting a Plk1-controlled polarity checkpoint in therapy-resistant glioblastoma-propagating cells. Cancer Res..

[18] Polyak D., Krivitsky A., Scomparin A., Eliyahu S., Kalinski H., Avkin-Nachum S., Satchi-Fainaro R. (2017). Systemic delivery of siRNA by aminated poly(α)glutamate for the treatment of solid tumors. J. Control. Release.

[19] Gibori H., Eliyahu S., Krivitsky A., Ben-Shushan D., Epshtein Y., Tiram G., Blau R., Ofek P., Lee J.S., Ruppin E. (2018). Amphiphilic nanocarrier-induced modulation of PLK1 and miR-34a leads to improved therapeutic response in pancreatic cancer. Nat. Commun.

[20] Scomparin A., Polyak D., Krivitsky A., Satchi-Fainaro R. (2015). Achieving successful delivery of oligonucleotides—From physico-chemical characterization to in vivo evaluation. Biotechnol. Adv..

[21] Ben-Shushan D., Markovsky E., Gibori H., Tiram G., Scomparin A., Satchi-Fainaro R. (2014). Overcoming obstacles in microRNA delivery towards improved cancer therapy. Drug Deliv. Transl. Res..

[22] Tiram G., Scomparin A., Ofek P., Satchi-Fainaro R. (2014). Interfering cancer with polymeric siRNA nanomedicines. J. Biomed. Nanotechnol..

[23] Maeda H., Wu J., Sawa T., Matsumura Y., Hori K. (2000). Tumor vascular permeability and the EPR effect in macromolecular therapeutics: A review. J. Control. Release.

[24] Markovsky E., Baabur-Cohen H., Eldar-Boock A., Omer L., Tiram G., Ferber S., Ofek P., Polyak D., Scomparin A., Satchi-Fainaro R. (2012). Administration, distribution, metabolism and elimination of polymer therapeutics. J. Contr. Release.

[25] Krivitsky A., Polyak D., Scomparin A., Eliyahu S., Ori A., Avkin-Nachum S., Krivitsky V., Satchi-Fainaro R. (2016). Structure–function correlation of aminated poly (α) glutamate as siRNA nanocarriers. Biomacromolecules.

[26] Krivitsky A., Krivitsky V., Polyak D., Scomparin A., Eliyahu S., Gibori H., Yeini E., Pisarevsky E., Blau R., Satchi-Fainaro R. (2018). Molecular weight-dependent activity of aminated poly (α) glutamates as siRNA nanocarriers. Polymers.

[27] Krivitsky A., Polyak D., Scomparin A., Eliyahu S., Ofek P., Tiram G., Kalinski H., Avkin-Nachum S., Gracia N.F., Albertazzi L. (2018). Amphiphilic poly(alpha)glutamate polymeric micelles for systemic administration of siRNA to tumors. Nanomedicine.

[28] Yeini E., Ofek P., Albeck N., Ajamil D.R., Neufeld L., Eldar-Boock A., Kleiner R., Vaskovich D., Koshrovski-Michael S., Israeli S.D. (2020). Targeting Glioblastoma: Advances in Drug Delivery and Novel Therapeutic Approaches. Adv. Ther..

[29] Muldoon L.L., Soussain C., Jahnke K., Johanson C., Siegal T., Smith Q.R., Hall W.A., Hynynen K., Senter P.D., Peereboom D.M. (2007). Chemotherapy delivery issues in central nervous system malignancy: A reality check. J. Clin. Oncol..

[30] Lorant D.E., Topham M.K., Whatley R.E., McEver R.P., McIntyre T.M., Prescott S.M., Zimmerman G.A. (1993). Inflammatory roles of P-selectin. J. Clin. Investig..

[31] Läubli H., Borsig L. (2010). Selectins Promote Tumor Metastasis.

[32] Yeini E., Ofek P., Pozzi S., Albeck N., Ben-Shushan D., Tiram G., Golan S., Kleiner R., Sheinin R., Dangoor S.I. (2021). P-selectin axis plays a key role in microglia immunophenotype and glioblastoma progression. Nat. Commun..

[33] Ferber S., Tiram G. (2017). Co-targeting the tumor endothelium and P-selectin-expressing glioblastoma cells leads to a remarkable therapeutic outcome. eLife.

[34] Wilkins P.P., Moore K.L., McEver R.P., Cummings R.D. (1995). Tyrosine sulfation of P-selectin glycoprotein ligand-1 is required for high affinity binding to P-selectin. J. Biol. Chem..

[35] Segal E., Pan H., Ofek P., Udagawa T., Kopeckova P., Kopecek J., Satchi-Fainaro R. (2009). Targeting angiogenesis-dependent calcified neoplasms using combined polymer therapeutics. PLoS ONE.

[36] Santana-Magal N., Farhat-Younis L., Gutwillig A., Gleiberman A., Rasoulouniriana D., Tal L., Netanely D., Shamir R., Blau R., Feinmesser M. (2020). Melanoma-Secreted Lysosomes Trigger Monocyte-Derived Dendritic Cell Apoptosis and Limit Cancer Immunotherapy. Cancer Res..

[37] Tiram G., Segal E., Krivitsky A., Shreberk-Hassidim R., Ferber S., Ofek P., Udagawa T., Edry L., Shomron N., Roniger M. (2016). Identification of dormancy-associated microRNAs for the design of osteosarcoma-targeted dendritic polyglycerol nanopolyplexes. ACS Nano.

[38] Dong H., Wang Q., Li N., Lv J., Ge L., Yang M., Zhang G., An Y., Wang F., Xie L. (2020). OSgbm: An online consensus survival analysis web server for Glioblastoma. Front. Genet..

[39] Kim J.-G., Sim S.J., Kim J.-H., Kim S.H., Kim Y.H. (2004). Synthesis and polymerization of methacryloyl-PEG-sulfonic acid as a functional macromer for biocompatible polymeric surfaces. Macromol. Res..

