# Supplementary material for: Sulfonated Amphiphilic Poly(α)glutamate Amine—A Potential siRNA Nanocarrier for the Treatment of Both Chemo-Sensitive and Chemo-Resistant Glioblastoma Tumors
Source: Pharmaceutics. 2021 Dec 20;13(12):2199. doi: 10.3390/pharmaceutics13122199 (PMC8705840; doi:10.3390/pharmaceutics13122199)
Supplement: Supplementary file 1 [file pharmaceutics-13-02199-s001.zip › pharmaceutics-1506919-supplementary.pdf]

*pharmaceutics*

MDPI

# Supplementary Materials: Sulfonated Amphiphilic Poly($\alpha$)glutamate Amine—A Potential siRNA Nanocarrier for the Treatment of Both Chemo-Sensitive and Chemo-Resistant Glioblastoma Tumors

**Adva Krivitsky, Sabina Pozzi, Eilam Yeini, Sahar Israeli Dangoor, Tal Zur, Sapir Golan, Vadim Krivitsky, Nitzan Albeck, Evgeny Pisarevsky, Paula Ofek, Asaf Madi and Ronit Satchi-Fainaro**

**A.** **B.**

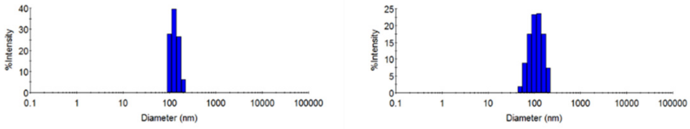


**C.**

| | N/P | Diameter by DLS [nm, Mean ± SD] | PDI [Mean ± SD] | Zeta potential [mV, Mean ± SD] |
|---|---|---|---|---|
| **APA:siGFP** | 5 | 115 ± 32 | 0.19 ± 0.26 | 0.52 ± 0.17 |

**D.**

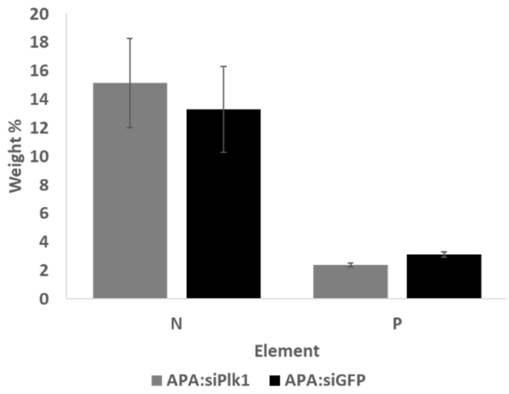


**Figure S1. Physicochemical characteristics of APA:siGFP polyplexes.** (**A**). APA:siPlk1 and (**B**). APA:siGFP representative histogram of the main population of polyplexes as obtained by Mobius DLS instrument. (**C**). A Table summarizing the hydrodynamic diameter, polydispersity index (PDI) and zeta potential of the main population of polyplexes, as obtained by Mobius and PALS instruments. (**D**). Elemental analysis demonstrating the weight percent of nitrogen (N) and Phosphorus (P) of dry samples of APA:siPlk and APA:siGFP polyplexes, as obtained by Energy-Dispersive X-Ray Spectroscopy (EDS) analysis.

**A.**

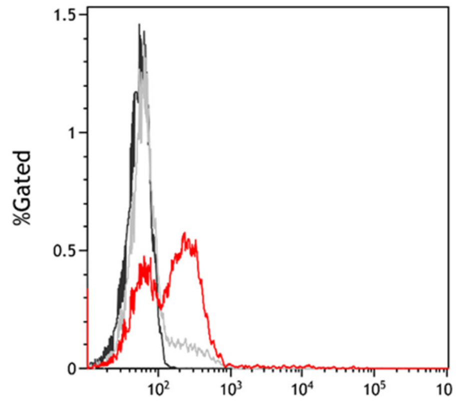


| | | % positive | X-Median |
|---|---|---|---|
| | Unstained | 1 | 55 |
| | 2D U251 | 12 | 65 |
| | 3D U251 | 56 | 164 |

**B.**

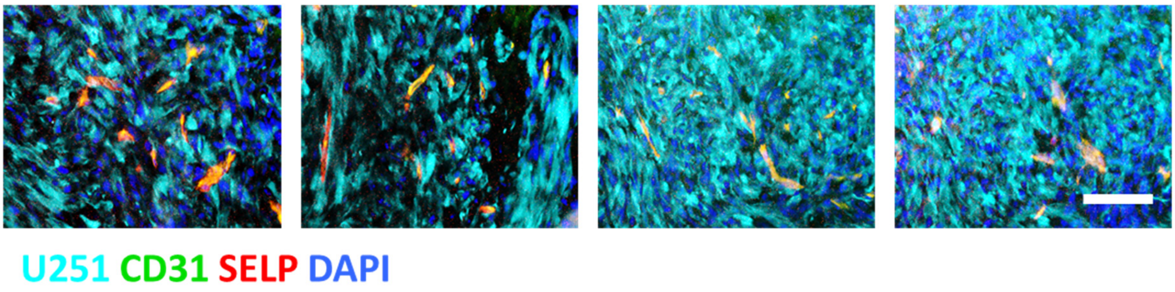


**Figure S2. SELP is overexpressed on GB cells and tumor endothelial cells in GB spheroids and *in vivo*.** (**A**). High expression of SELP on U251 spheroids (3D) compared with tissue culture (2D) as obtained by fluorescence-activated cell sorting (FACS). (**B**). Co-localization of SELP with CD31 in U251 intracranial tumors (scale= 100 µm).

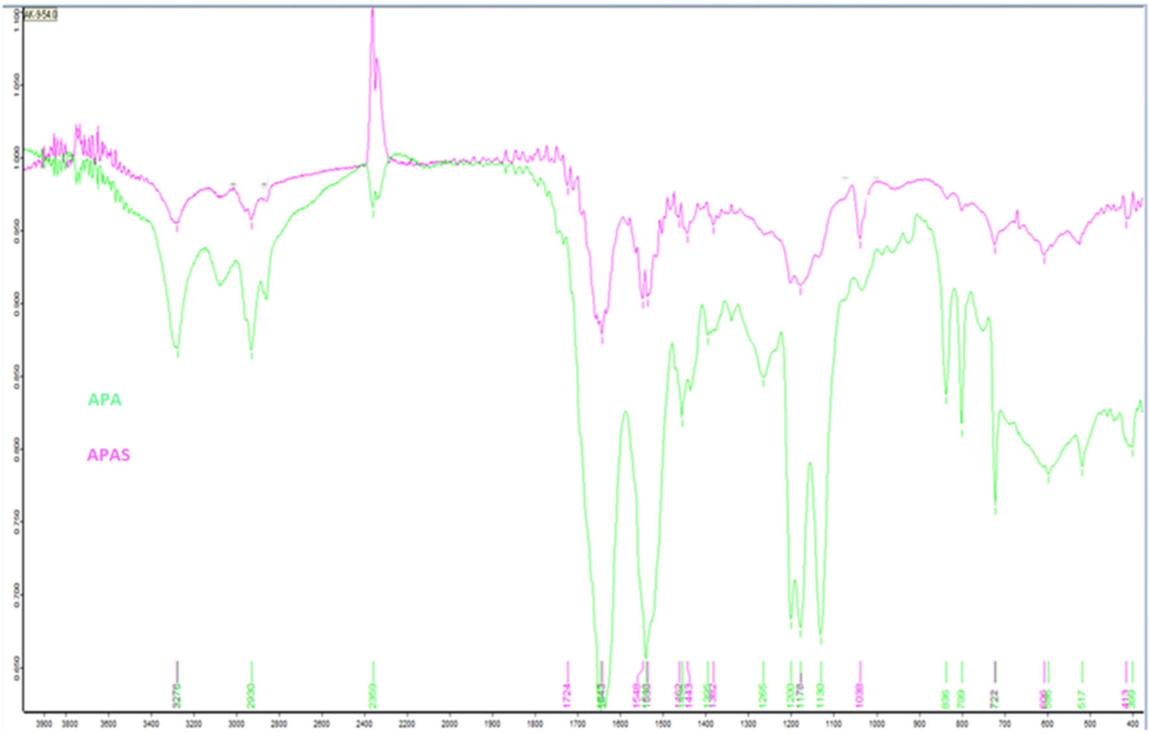


**Figure S3.** IR spectrum of APA and APAS.

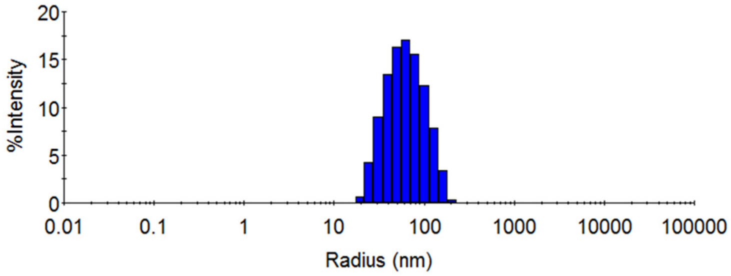


**Figure S4.** APAS:siPlk1 representative histogram of the main population of polyplexes as obtained by Möbius™ DLS instrument.

**A.**

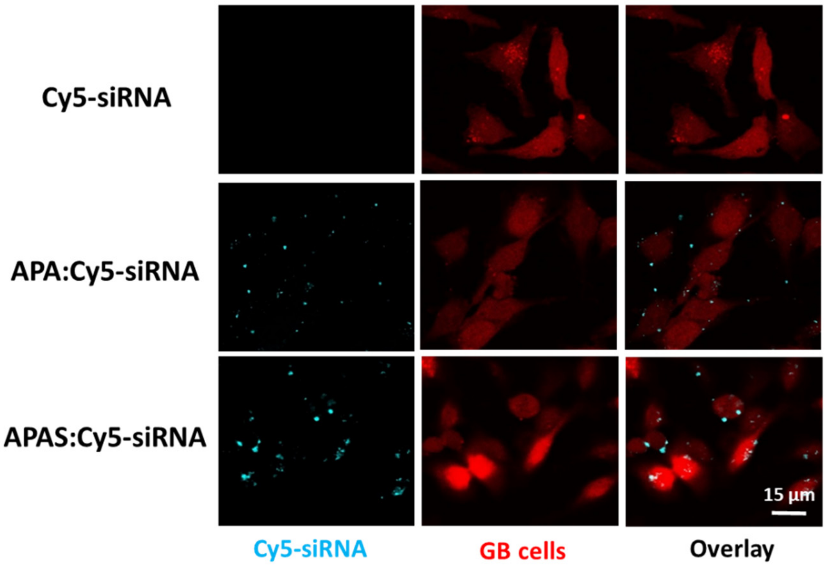


**B.**

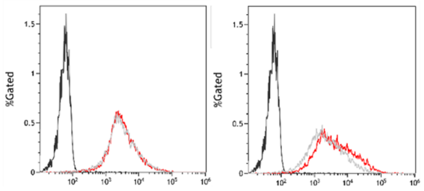


| | | APA:Cy5-siRNA | APAS:Cy5-siRNA |
|---|---|---|---|
| | Unstained | 58 | 58 |
| | Polyplex | 2931 | 3536 |
| | Polyplex + SELP inhibitor | 2847 | 2016 |

**Figure S5. Sulfonate-mediated cell internalization via SELP.** (**A**). Sulfonate modification enhanced the internalization of Cy5-siRNA carrying polyplexes into U251 cells. (**B**). Inhibition of SELP reduced internalization of APAS:Cy5-siRNA polyplexes into GL261 spheroids, but did not alter the internalization of APA:Cy5-siRNA polyplexes, as obtained by FACS.
